# Neuroinflammation is not a Prerequisite for Diabetes-induced Tau Phosphorylation

**DOI:** 10.3389/fnins.2015.00432

**Published:** 2015-11-09

**Authors:** Judith M. van der Harg, Leslie Eggels, Silvie R. Ruigrok, Jeroen J. M. Hoozemans, Susanne E. la Fleur, Wiep Scheper

**Affiliations:** ^1^Department of Genome Analysis, Academic Medical Center, University of AmsterdamAmsterdam, Netherlands; ^2^Departments of Functional Genomics and Molecular and Cellular Neuroscience, Center for Neurogenomics and Cognitive Research, Neuroscience Campus Amsterdam, VU University AmsterdamAmsterdam, Netherlands; ^3^Department of Endocrinology and Metabolism, Academic Medical Center, University of AmsterdamAmsterdam, Netherlands; ^4^Department of Pathology, Neuroscience Campus Amsterdam, VU University Medical CenterAmsterdam, Netherlands; ^5^Department of Clinical Genetics and Alzheimer Center, VU University Medical CenterAmsterdam, Netherlands

**Keywords:** neuroinflammation, Alzheimer's disease, Diabetes Mellitus, phosphorylated tau, cortex, hippocampus

## Abstract

Abnormal phosphorylation and aggregation of tau is a key hallmark of Alzheimer's disease (AD). AD is a multifactorial neurodegenerative disorder for which Diabetes Mellitus (DM) is a risk factor. In animal models for DM, the phosphorylation and aggregation of tau is induced or exacerbated, however the underlying mechanism is unknown. In addition to the metabolic dysfunction, DM is characterized by chronic low-grade inflammation. This was reported to be associated with a neuroinflammatory response in the hypothalamus of DM animal models. Neuroinflammation is also implicated in the development and progression of AD. It is unknown whether DM also induces neuroinflammation in brain areas affected in AD, the cortex and hippocampus. Here we investigated whether neuroinflammation could be the mechanistic trigger to induce tau phosphorylation in the brain of DM animals. Two distinct diabetic animal models were used; rats on free-choice high-fat high-sugar (fcHFHS) diet that are insulin resistant and streptozotocin-treated rats that are insulin deficient. The streptozotocin-treated animals demonstrated increased tau phosphorylation in the brain as expected, whereas the fcHFHS diet fed animals did not. Remarkably, neither of the diabetic animal models showed reactive microglia or increased GFAP and COX-2 levels in the cortex or hippocampus. From this, we conclude: 1. DM does not induce neuroinflammation in brain regions affected in AD, and 2. Neuroinflammation is not a prerequisite for tau phosphorylation. Neuroinflammation is therefore not the mechanism that explains the close connection between DM and AD.

## Introduction

Alzheimer's disease (AD) is a multifactorial neurodegenerative disorder characterized by aggregation of Aβ and abnormal phosphorylation and aggregation of tau (Grundke-Iqbal et al., 1986; Selkoe, 1991). Neuroinflammation is associated with the development and progression of AD. Different genome-wide association studies identified genes involved in the innate immune system as risk factors for AD; *CR1, CD33*, and *TREM2*, which are all expressed in microglia (Bertram et al., 2008; Harold et al., 2009; Lambert et al., 2009; Jonsson et al., 2013). Moreover, microglia are activated early in AD pathology (Hoozemans et al., 2005). Microglial activation is already observed in patients with mild cognitive impairment (MCI, Okello et al., 2009). Interestingly, the density of activated microglial correlates inversely with cognitive performance of AD patients (Combs, 2009). These data strongly indicate the involvement of neuroinflammation in AD pathogenesis.

However, the exact underlying pathomechanism of neuroinflammation in AD pathology is complex. Neuroinflammation influences both the clearance and production of toxic tau and Aβ species. Proinflammatory cytokines reduce the clearance capacity of glial cells thereby resulting in a disbalance of production and clearance of accumulated proteins (Lee and Landreth, 2010). In addition, BACE1 activity is increased by inflammation *in vitro* and is reduced after using nonsteroidal anti-inflammatory drugs in AD mice (Sastre et al., 2003, 2006; Lee et al., 2008). Tau phosphorylation is increased by a shift in the balance of tau kinase and phosphatase activity (Arnaud et al., 2006). The activity of the tau kinases GSK3β, Cdk5, and p38-MAPK is increased upon inflammation. In addition, a different pathway of inducing tau pathology by neuroinflammation was described by Arnaud et al. (2009) showing that inflammation leads to tau cleavage into an aggregation-prone form known to seed tau aggregation.

Epidemiological studies show that Diabetes Mellitus (DM) is a risk factor for AD and that the incidence of AD is higher in people with DM (Biessels et al., 2006; Kopf and Frölich, 2009). Moreover, DM is associated with higher risk for MCI (Luchsinger et al., 2007). DM is characterized by marked high levels of blood glucose and occurs in two forms: type 1 DM (T1DM), which results from insulin deficiency, and type 2 DM (T2DM) which starts with overproduction of insulin due to insulin resistance and over time results like T1DM in extreme hyperglycemia. In transgenic AD models, both insulin deficiency and insulin resistance exacerbate tau pathology (Ke et al., 2009; Park, 2011). Interestingly, various studies show induction of endogenous tau phosphorylation in the brains of T1DM animal models (reviewed by Park, 2011; El Khoury et al., 2014). An increased level of endogenous tau phosphorylation is also reported in some animals on high-caloric diet that develops insulin resistance. However, this is not consistently observed (Table 1).

**Table 1:** **Overview of tau phosphorylation in diet-induced diabetic models**.

**Treatment**	**Species**	**Duration**	**Tau phosphorylation**	**Publication**
**DIET MODEL**				
HF	Mice	12–16 weeks	↔	Becker et al., 2012
HF	Mice	16 weeks	↔	Moroz et al., 2008
HF	Mice	32 weeks	↓	To et al., 2011
HF	Mice	18 weeks	↔	Ramos-Rodriguez et al., 2013
HF THY-Tau22 or WT	Mice	5 months	↑ THY-Tau22, ↔ WT	Leboucher et al., 2013
HF	Rat	12 weeks	↔	McNeilly et al., 2012
HF	Rat	8 weeks	↑	Zhang et al., 2010b
HF + HC	Rat	8 weeks	↑	Bhat and Thirumangalakudi, 2013
**DIET + STZ MODEL**				
HF + HS + HP + STZ	Rat	16 weeks	↑	Yang et al., 2013
HF + STZ	Rat	4 weeks	↑	Zhang et al., 2010a

*HF, high-fat diet; WT, wild type; HC, high-cholesterol diet; HS, high-sugar diet; HP, high-protein diet; ↑, increase of tau phosphorylation; ↓, decrease of tau phosphorylation; ↔, no change in tau phosphorylation*.

Interestingly, DM is characterized by low-grade systemic inflammation. Inflammation has been implicated in the progression and peripheral complications of both T1DM and T2DM (King, 2008; Gustafson, 2010; Vykoukal and Davies, 2011). This peripheral inflammation can be accompanied by neuroinflammation in specific regions of the central nervous system. Reactive glial cells and activation of different cytokines are reported in the hypothalamus of insulin deficient (Luo et al., 2002) as well as insulin resistant animals and in obese humans (Thaler et al., 2012). However, the adverse effects of insulin deficiency or insulin resistance on regions of the brain involved in cognition (cortex and hippocampus) are hardly investigated. Therefore, we investigated whether neuroinflammation could be the mechanistic trigger to induce tau pathology in the brain of DM animals. Two distinct diabetic animal models were used to study neuroinflammation in the cortex and the hippocampus, brain areas primarily affected in AD. The first model mimics T1DM by destroying the pancreatic β cells with streptozotocin (STZ) resulting in insulin deficiency and extreme hyperglycemia (Qu et al., 2011). In the second model, rats are fed a free-choice high-fat high-sugar (fcHFHS) diet for 10 weeks to model obesity-induced insulin resistance. Previously we showed that rats have increased body weight, slight hyperglycemia, hyperinsulinemia, glucose intolerance and a diminished insulin response to a glucose load after a 4-week fcHFHS diet (la Fleur et al., 2011). In this study, we investigated whether inflammation, a common dominator in both insulin deficient and insulin resistant animals, can lead to tau phosphorylation using these two animal models.

## Materials and methods

### Animals

This study was performed with male Wistar rats (250–350 g; Charles River, Sulzfeld, Germany). Rats were individually housed under a 12:12 h light/dark cycle (lights on 07.00 h) at 21–23°C with *ad libitum* access to standard chow (Special Diets Services, Essex, United Kingdom) and tap water. Two distinct diabetic animal models were used: an insulin resistance diet model and insulin deficient model. Rats on fcHFHS diet received in addition to the control groups a dish of saturated fat [beef tallow (Ossewit/Blanc de Boeuf, Vandermoortele, Belgium)] and a bottle of 30% sugar water (1 M sucrose mixed from commercial grade sugar and water) in the cage for 1 week (*n* = 6), 4 weeks (*n* = 6), or 10 weeks (*n* = 9). For the second diabetic model, T1DM, rats received under anesthesia of isoflurane subcutaneous an injection of STZ (experimental group) (Sigma Aldrich, St. Louis, MO, USA; 65 mg/kg in 0.3 ml citrate buffer pH 4.2) or vehicle (citrate buffer). Animals were sacrificed 20 days after injection (*n* > 5 animals per group). For western blot analysis, animals were decapitated under CO_2_/O_2_ and brains were quickly removed and collected on dry ice before frozen in −80°C. Subsequently, hippocampus and cortex were dissected for protein lysates. For immunohistological analysis, animals were transcardially perfused with phosphate-buffered saline (PBS) followed by 4% paraformaldehyde under Nembutal anesthetics (120 mg/kg). Brains were embedded in paraffin. During the experiment body weight, food and water intake was measured 3 times a week. Glucose and insulin levels were not determined since the therefore required clamp studies or fasting of the animals both have a profound effect on the brain and could interfere with our results (Faggioni et al., 2000; Rummel et al., 2010; Lavin et al., 2011; Fuente-Martin et al., 2012; Bowe et al., 2014; Routh et al., 2014; Vasconcelos et al., 2014). However, previously fasting hyperinsulinemia and slight increases in glucose levels have been shown in the fcHFHS model used in this study (la Fleur et al., 2010, 2011; Harris and Apolzan, 2012). In STZ model trunk blood was collected to measure plasma glucose concentration with the Biosen (EKF diagnostics, Cardiff, UK) following the assay protocol. A significant increase in plasma glucose levels without fasting of STZ-treated animals compared to control rats was detectable due to the extreme hyperglycemia levels of STZ-treated rats (6.28 ± 0.44 mmol/L basal glucose levels in control rats vs. 20.9 ± 4.95 mmol/L basal glucose levels in STZ-treated rats). The experiment was approved by the Committee for Animal Experimentation of the Academic Medical Center of the University of Amsterdam, the Netherlands.

### SDS-PAGE and western blotting

Brain lysates were homogenized with a seirin needle and incubated for 30 min on ice in RIPA buffer (50 mM TrisHCl, 150 mM NaCl, 1% NP-40, 0.5% Sodium deoxycholate, 0.1% SDS, 2 mM EDTA) supplemented with protease and phosphatase inhibitors. Subsequently, brain lysates were centrifuged for 15 min at 20.000 × g at 4°C. Supernatant protein content was determined by BCA protein assay kit (Pierce, Rockford, IL, USA). Equal amounts of protein were loaded on 10% polyacrylamide gels and blotted onto nitrocellulose membrane (Millipore, Billerica, MA, USA). Blots were pre-incubated with 5% bovine serum albumin (BSA; Boehringer, Mannheim, Germany) in TBS-T [0.05% Tween-20 in Tris buffered saline (TBS)] for 60 min at room temperature and subsequently incubated at 4°C overnight with primary antibodies. Membranes were washed 3 × 10 min in TBS-T and subsequently incubated with species-specific secondary antibodies conjugated to horseradish peroxidase (dilution 1:2000, Dako, Glostrup, Denmark). Reactive protein bands were visualized using LumiLightPLUS Western blotting substrate (Roche Applied Science). Results were analyzed using Advanced Image Data Analyzer software (Raytest, Straubenhardt, Germany) version 3.44.035 and using Image Studio Version 2.0 software (Li-cor, Lincoln, NE, USA). Brain samples from the experimental and the control group were all loaded on the same SDS-PAGE gel. The primary antibodies and their dilution factors are listed in Table 2.

**Table 2:** **Primary antibodies**.

**Antibody**	**Species**	**Dilution**	**Company**
**ANTIBODIES FOR WESTERN BLOT ANALYSES**			
eEF2α	Rabbit	1:1000 in 5% BSA/TBS-T	Cell Signal, USA
p-tau (Ser^396^)	Rabbit	1:1000 in 5% BSA/PBS-T	Cell Signal, USA
Actin	Mouse	1:1000 in 5% BSA/TBS-T	Cell Signal, USA
GFAP	Rabbit	1:1000 in 5% BSA/TBS-T	DAKO, DE
COX-2	Mouse	1:1000 in 5% BSA/TBS-T	Cayman chemical, USA
**ANTIBODIES FOR IMMUNOHISTOCHEMISTRY**			
AT8	Mouse	1:200	Pierce, Rockford, USA
IBA-1	Rabbit	1:1000	Novus Biologicals, USA

### Immunohistochemistry

Brains were cut into 5 μm sagittal sections. The sections were immersed in 0.3% H_2_O_2_ in TBS for 30 min to quench endogenous peroxidase activity. Sections were treated with 10 mmol/l, pH 6.0, sodium citrate buffer (AT8) or with 10 mM Tris and 1 mM EDTA buffer, pH 9.0 (IBA-1) for 10 min at 99°C for antigen retrieval and subsequently incubated with primary antibodies at 4°C overnight. Antibodies (Table 2) were diluted in TBS containing 0.5% triton-X-100. Negative controls for all immunostainings were generated by omission of primary antibodies. Sections were washed with TBS and subsequently incubated for 120 min with undiluted EnVision/HRP anti-rabbit/mouse (Dako, Hamburg, Germany). Color was developed using 3, 3′-diaminobenzidine (EnVision detection system/HRP 1:50, DakoCytomation, Glostrup, Denmark) as chromogen. Sections were counterstained with haematoxylin and mounted using Depex (BDH Laboratories Supplies, East Grinstead, UK).

### Statistical analysis

GraphPad Prism software was used for graphs and statistical analysis. All data are compared to the control group. Two-sided unpaired Student's *t*-test was used for single statistical comparison. No significance difference was defined as *P* > 0.05.

## Results

**Figure 1:**
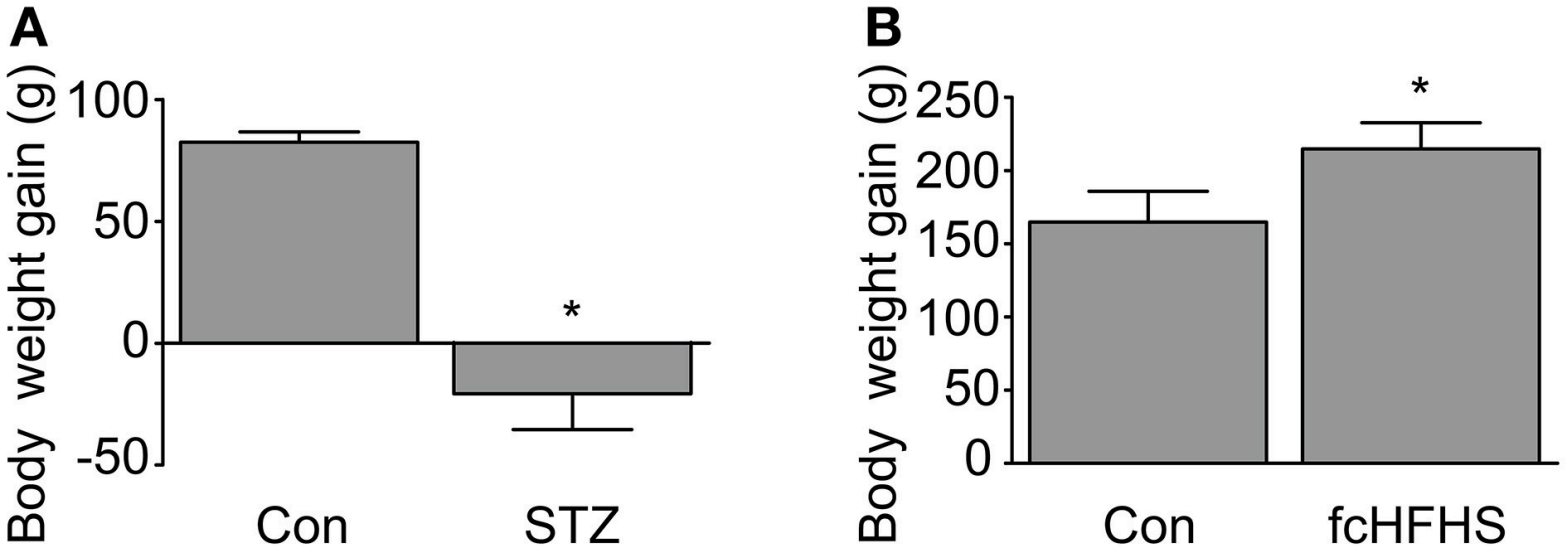
**Body weight gain in diabetic rats**. Body weight gain 20 days after citrate buffer (con) or STZ injection is shown **(A)**. Data is presented as mean ± *SD* of *n* = 6 animals per group. STZ treatment results in loss of approximately 20 grams (g) of body weight from the start of the experiment. Body weight gain after 10-weeks standard chow diet (con) or fcHFHS diet **(B)** are shown as mean ± *SD* of *n* = 9 animals per group. Rats on fcHFHS diet gain on average 50 g more weight than rats on standard chow diet. (^*^p < 0.01).

**Figure 2:**
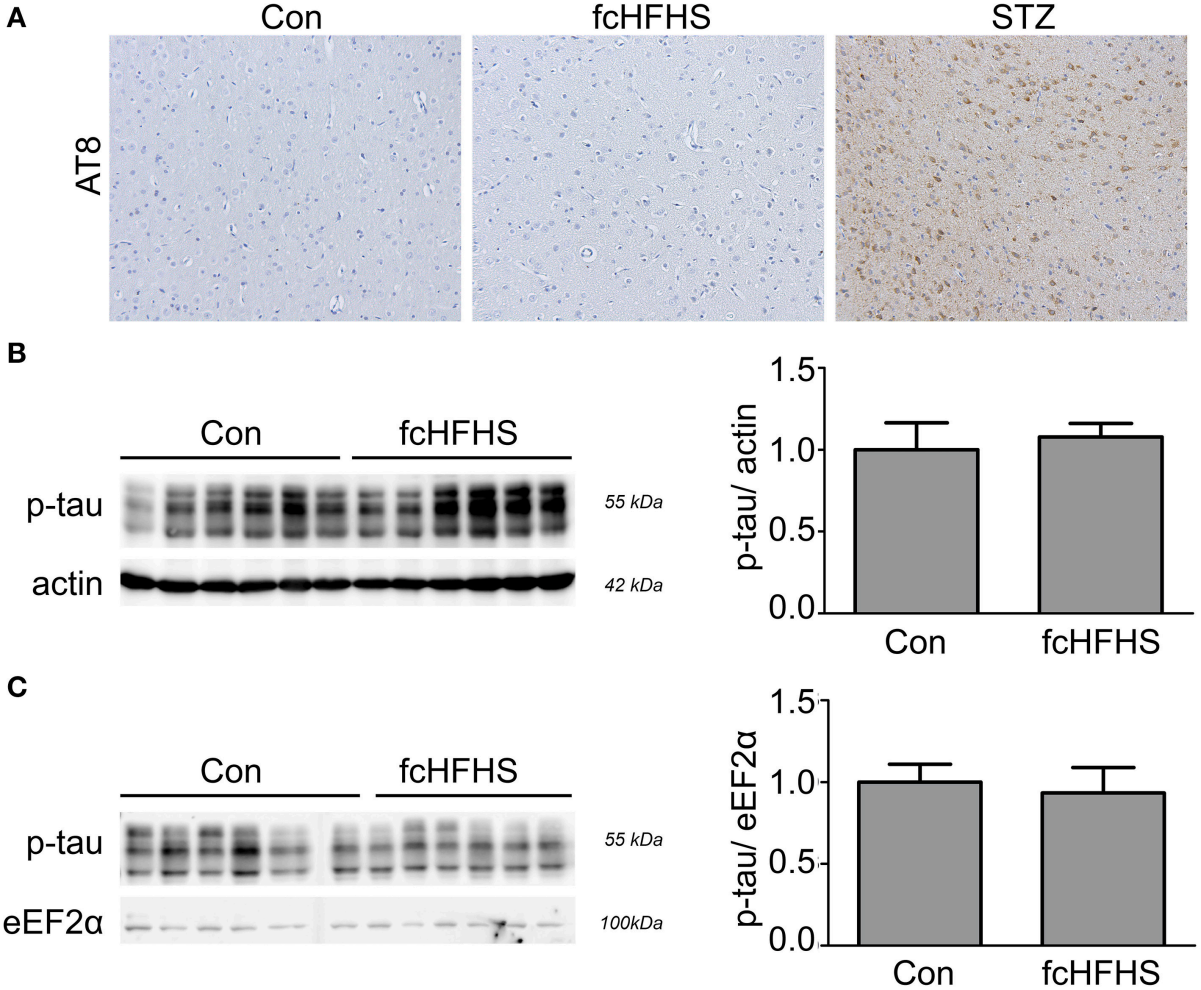
**Tau phosphorylation in diabetic rats**. Sagittal brain sections of control rats (con), rats on 10-weeks fcHFHS diet and STZ-treated rats were stained for tau using the AT8 antibody. Representative immunohistochemical images of the cortex are shown **(A)**. Tau phosphorylation was observed in STZ-treated rats, but no positive reactivity was found in the animals on the fcHFHS diet. Western blot analyses of hippocampus protein lysates of animals on fcHFHS diet for 4 weeks **(B)** or 1 week **(C)** did not show an increase in tau Ser396 phosphorylation (p-tau) compared to control animals on standard chow diet (con). Quantification of western blot is presented as mean ± SEM of *n* = 6 animals per group.

To investigate whether there is neuroinflammation in AD-affected brain areas as a complication of diabetes we used a STZ model and fcHFHS diet model. In both diabetic models body weight gain was measured at the end of the experiment to ensure effectiveness of the treatment. As expected, STZ treatment reduced body weight (Figure 1A). In line with a 4-week fcHFHS diet, body weight gain was significantly increased after a 10-week fcHFHS diet compared to standard chow diet (Figure 1B). We first investigated endogenous levels of phosphorylated tau in the brain of both diabetic models. Immunohistochemistry for phosphorylated tau using the AT8 antibody on brain tissue of rats after 10-weeks fcHFHS diet showed no positive reactivity (Figure 2A). In contrast, the STZ model showed increased tau phosphorylation in the cortex, as previously reported in several studies (Park, 2011; El Khoury et al., 2014). To ascertain that tau phosphorylation did not occur as an adaptive response just after the start of the fcHFHS diet that disappeared after adjustment to the diet, tau phosphorylation was studied at earlier time points. Western blot analyses of hippocampal protein lysates of rats fed a fcHFHS diet for 4-weeks (Figure 2B) and 1-week (Figure 2C) were performed. There was no increased tau phosphorylation at Ser396 observed after 4-week or 1-week diet. As a positive control, western blot analyses of tau phosphorylation at Ser396 were performed in STZ-treated rats showing increased levels of phosphorylated tau in the STZ model using this method as well (Supplementary Figure 1). These results demonstrate that -in contrast to the STZ model- the fcHFHS diet model does not induce tau phosphorylation.

**Figure 3:**
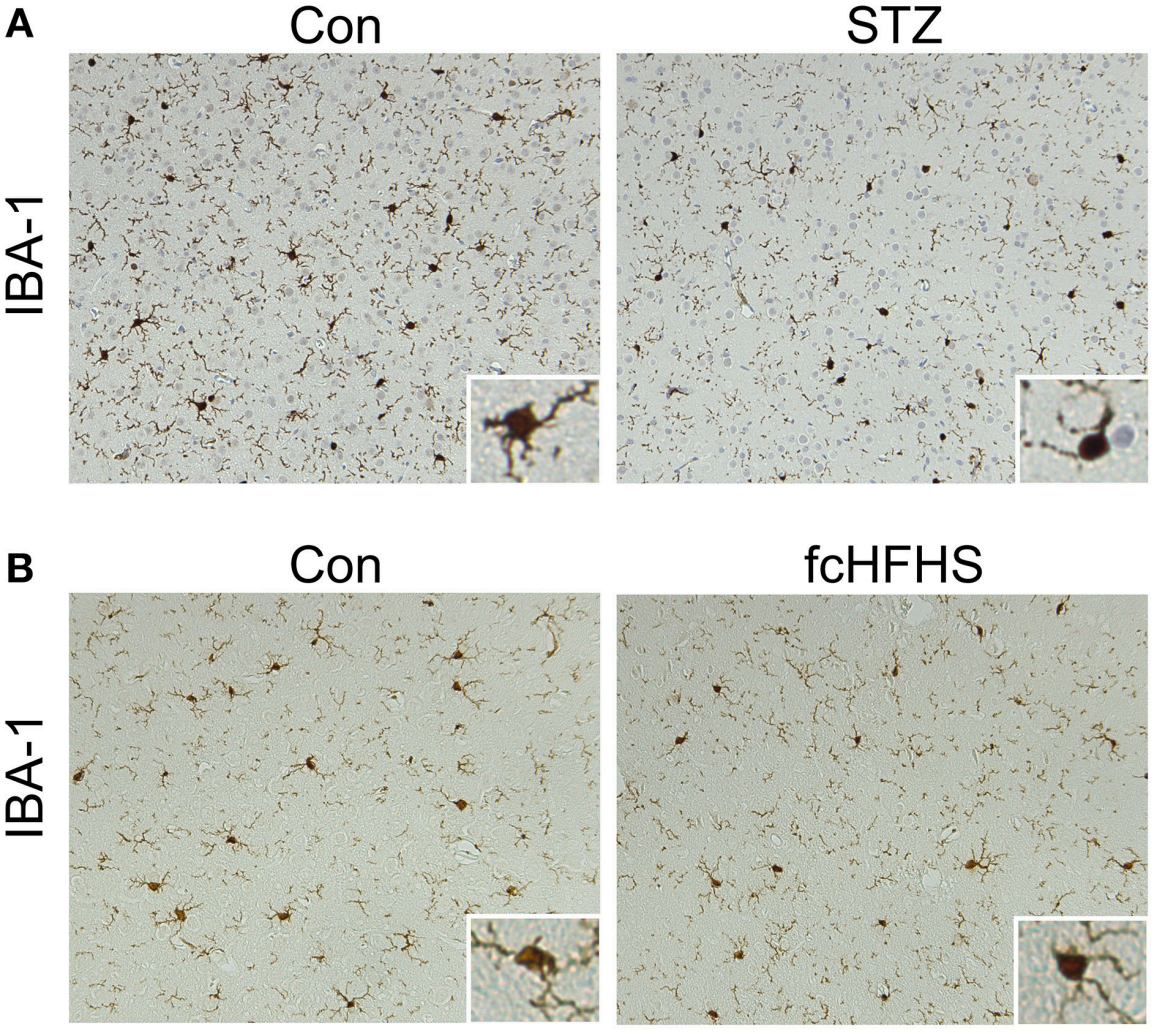
**No reactive microglia in diabetic rat cortex**. IBA-1 immunohistochemistry was performed on sagittal brain sections of rats 20 days after injection of citrate buffer (con) or STZ **(A)** and of rats on a 10-week standard chow diet (con) or fcHFHS diet **(B)**. Representative images of the cortex are showed of *n* = 9 animals per diet group and *n* = 6 animals per injected group. No difference in microglial morphology is observed compared to control group either in STZ-treated rats or in rats on fcHFHS diet.

**Figure 4:**
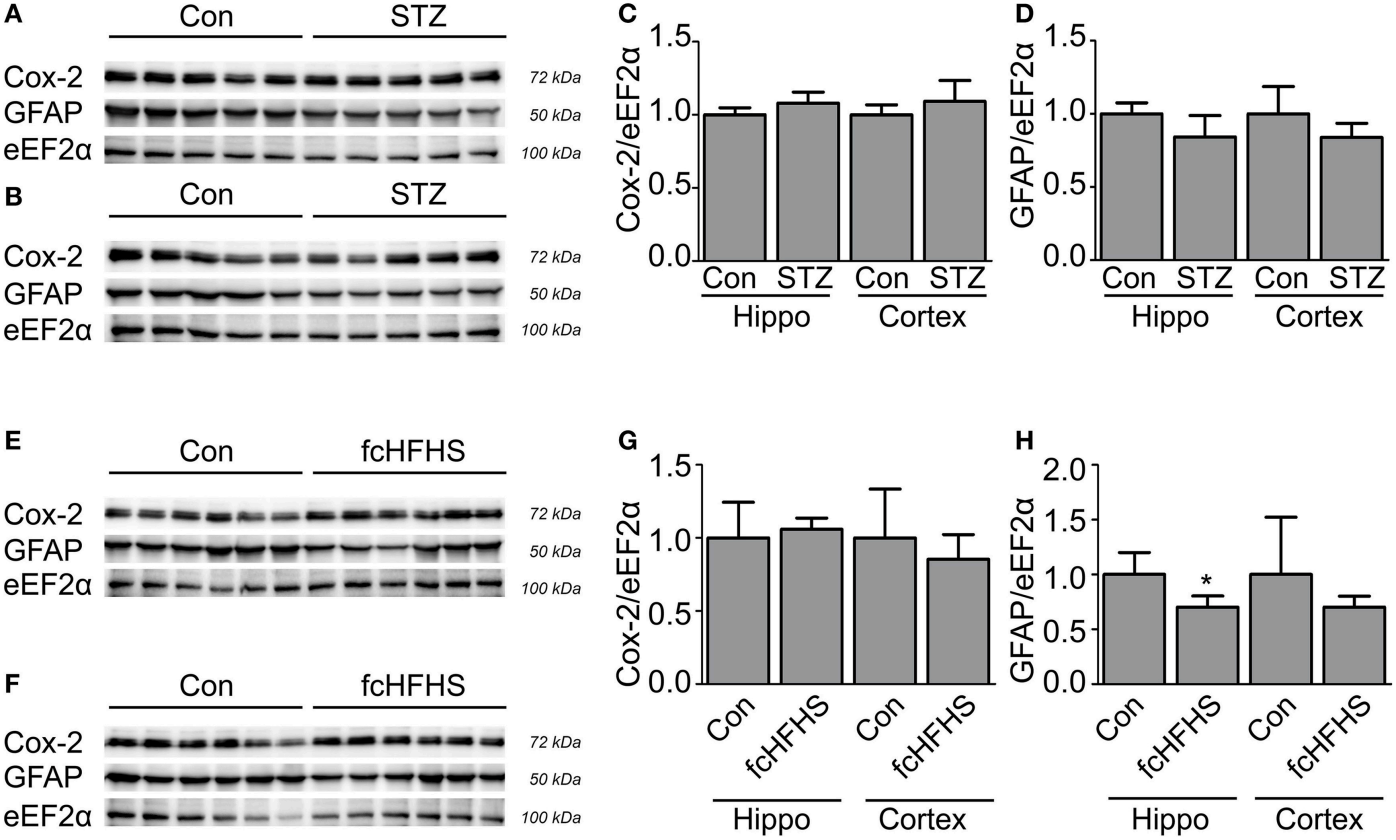
**No increased COX-2 and GFAP levels in diabetic rat cortex or hippocampus**. Western blot analyses of protein lysates of STZ-treated animals **(A–D)** and animals on 4-week fcHFHS diet **(E–H)** of the hippocampus (Hippo) **(A,E)** and cortex **(B,F)** were performed. Quantification of COX-2 levels in STZ model **(C)** and diet model **(G)** did not show a difference compared to the control. Quantification of GFAP levels in STZ-treated animals did not show a change **(D)**. GFAP levels after 4-week diet even showed a decrease **(H)**. Quantification of western blot is presented as mean ± *SD* of *n* = 5 or 6 animals per group. (^*^p < 0.01).

The differential effects of the STZ treatment and the fcHFHS diet on tau phosphorylation provides the opportunity to study whether neuroinflammation is inextricably linked to increased tau phosphorylation. Inflammation has been associated with the progression and peripheral complications of both T1DM and T2DM (King, 2008; Gustafson, 2010; Vykoukal and Davies, 2011). It is not known whether this is associated with an inflammatory response in the brain as well, therefore, we investigated neuroinflammation in the cortex and the hippocampus in both models. IBA-1 staining was performed to study the morphology of microglia. Reactive microglia are clustered and characterized by shorter and thicker processes. We did not observe a difference in microglial morphology in the brains 20 days after STZ injection (Figure 3A) nor did we observe a difference in microglial morphology in animals after a 10-week fcHFHS diet compared to standard chow diet (Figure 3B). Therefore, the cortex and hippocampus did not show differences in reactive microglia. To exclude that a moderate neuroinflammatory response is induced cyclooxygenase-2 (COX-2) was studied. COX-2 is upregulated in activated microglia and dynamically regulated by pro-inflammatory signals (Minghetti et al., 1999). Western blot analyses of COX-2 in STZ-treated animals (Figures 4A–C) and in animals after 4-weeks fcHFHS diet (Figures 4E–G) did not show upregulation of COX-2 in the cortex and the hippocampus. Finally, glial fibrillary acidic protein (GFAP) was studied as marker for reactive astrocytes. Also GFAP levels in STZ-treated animals (Figures 4A,B,D) and in animals after 4-weeks fcHFHS diet (Figures 4E,F,H) were not increased in the cortex and the hippocampus. A 4-week fcHFHS diet even resulted in a significant decrease of GFAP levels. These data demonstrate that neuroinflammation is not a complication of diabetes in brain areas affected in AD. Moreover, increased tau phosphorylation can occur independently of neuroinflammation in insulin deficient and insulin resistant animal models.

## Discussion

The present study demonstrates absence of reactive microglia and increased levels of GFAP and COX-2 in the hippocampus and the cortex of two distinct diabetic animal models. This indicates that DM does not directly lead to neuroinflammation in AD-affected brain areas. An increased level of endogenous tau phosphorylation was observed in STZ-treated rats demonstrating that neuroinflammation is not a prerequisite for diabetes-induced tau phosphorylation. The absence of increased tau phosphorylation in our fcHFHS diet model is in accordance with the literature (Table 1). Other studies using a high-fat diet also did not observe an increased level of endogenous tau phosphorylation (Moroz et al., 2008; To et al., 2011; Becker et al., 2012; McNeilly et al., 2012; Leboucher et al., 2013; Ramos-Rodriguez et al., 2013). The studies that did observe diet-induced tau phosphorylation, used a tau transgenic mouse or combined the diet with a STZ treatment at the end of the experiment to mimic the hyperglycemic state of T2DM (Zhang et al., 2010a; Leboucher et al., 2013; Yang et al., 2013). Only one study using a high-fat diet with hyperglycemia levels to the extent observed in STZ-induced animals and one study with a high-fat and high-cholesterol diet for 2 months reported an increased level of endogenous phosphorylated tau (Zhang et al., 2010b; Bhat and Thirumangalakudi, 2013). Overall, this suggests that insulin resistance due to a high-caloric diet is not sufficient to induce tau phosphorylation. Nevertheless, the observation that both diabetic models regardless of tau phosphorylation showed no neuroinflammation indicates that neuroinflammation is not the mechanistic trigger to induce tau phosphorylation in the diabetic brain.

Inflammation was reported in the hypothalamus of STZ-treated animals and animals on a high-caloric diet (Luo et al., 2002; Thaler et al., 2012). Microglial activation in the hypothalamus, particularly in the arcuate nucleus, is already observed after 1 day. The permeable blood brain barrier of this area may explain the neuroinflammatory response whereas other brain areas are less accessible for influences from the periphery (Davidson et al., 2012). Indeed, bypassing of the blood brain barrier by direct intracerebroventricular injection of STZ results in glial activation and neuroinflammation in the hippocampus and the cortex (Prickaerts et al., 1999; Chen et al., 2013). This suggests that the adverse effects of STZ resulting in neuroinflammation do not reach the AD-affected areas after intraperitoneal STZ injection. Finally, some studies show increased glial activation in the hippocampus or the cortex after prolonged treatment. One study showed only astrocytic activation in the cortex after 21-weeks of 41%-fat diet and extensive neuroinflammation after 21-weeks of 60%-fat diet (Pistell et al., 2010). In addition, increased astrocytic reactivity was found in the hippocampus of mice 4-weeks after a single intraperitoneal STZ injection (Saravia et al., 2002). This indicates that neuroinflammation in AD-affected brain areas upon diet and STZ treatment occurs only upon prolonged and extensive treatment. Since tau phosphorylation is already observed 20 days after STZ treatment, neuroinflammation is also unrelated to increased phosphorylated tau levels in these studies.

Although we demonstrate that neuroinflammation is not a prerequisite for increased tau phosphorylation, our data do not exclude that peripheral inflammation could have an effect on the brain resulting in increased tau phosphorylation independent of neuroinflammation. In addition, DM may alter the neuroinflammatory response or even trigger neuroinflammation after a priming stimulus and thus facilitate AD pathology. As mentioned above there is a body of evidence to suggest that neuroinflammation plays a role in the progression of AD (Hoozemans and O'Banion, 2005; Bertram et al., 2008; Combs, 2009; Harold et al., 2009; Lambert et al., 2009; Okello et al., 2009; Jonsson et al., 2013). Interestingly, in an AD animal model for Aβ pathology neuroinflammation occurred after the increase of Aβ levels, but before the onset of plaque formation (Hanzel et al., 2014). Indeed, neuropathological and experimental studies demonstrate that Aβ can activate the immune system including the pro-inflammatory cytokine cascade (Eikelenboom et al., 2012; Heneka et al., 2014). In addition, in transgenic animals modeling tau pathology neuroinflammation was observed after initiation of tau pathology and in turn, tau phosphorylation was exacerbated by lipopolysacharide injections into the brain (Zilka et al., 2009; Lee et al., 2010). This indicates that neuroinflammation is a secondary response upon increased Aβ and phosphorylated tau. Subsequently, neuroinflammation can increase Aβ production and tau phosphorylation resulting in a vicious cycle that accelerates AD pathology. This is in accordance with our observations in the T1DM model, which mimics the early phase of tau pathology and shows no neuroinflammation.

As neuroinflammation is not the trigger for increased tau phosphorylation the question remains which mechanism induces endogenous tau phosphorylation in the diabetic brain. Interestingly, dysfunction in insulin signaling has been reported in post-mortem AD brain material and in animal models of AD (Steen et al., 2005; Lester-Coll et al., 2006; de la Monte, 2009; Moloney et al., 2010; Talbot et al., 2012). Since increased tau phosphorylation was not observed in both the STZ model and fcHFHS diet model, insulin deficiency and insulin resistance may have a differential effect on tau phosphorylation. In addition, it should be mentioned that the STZ model has extreme hyperglycemia to the extent of T1DM that may contribute to tau phosphorylation whereas high-caloric diet models show slight hyperglycemia not comparable with the final state of T2DM. Finally, we previously reported increased tau phosphorylation upon metabolic stress in a physiological hypometabolic model *in vivo* indicating that also metabolic stress in DM could be the trigger to induce tau phosphorylation (van der Harg et al., 2014). Further research is needed to better understand the differences of insulin deficiency and insulin resistance on tau phosphorylation and to find the underlying mechanism for diabetic-induced tau phosphorylation.

In conclusion, our data demonstrate that increased tau phosphorylation occurs independently of neuroinflammation. Eliminating neuroinflammation as causal factor facilitates further elucidation of the complex connection between DM and AD and helps to put the multiple underlying mechanisms contributing to AD in an integrated framework (van Dijk et al., 2015). Since the incidence of both DM and AD is increasing due to the aging population, better understanding of the connection between these diseases is crucial. Further research is necessary to discover the mechanistic trigger for increased tau phosphorylation in the diabetic brain.

## Author contributions

All authors substantial contributed to the conception and design of the work and the critically drafting of the manuscript. All authors approved the manuscript to be published and agreed to be accountable for the work.

### Conflict of interest statement

The authors declare that the research was conducted in the absence of any commercial or financial relationships that could be construed as a potential conflict of interest.
