# Supplementary material for: Neuroinflammation is not a Prerequisite for Diabetes-induced Tau Phosphorylation
Source: Front Neurosci. 2015 Nov 9;9:432. doi: 10.3389/fnins.2015.00432 (PMC4637426; doi:10.3389/fnins.2015.00432)
Supplement: Supplementary file 1 [file Image1.PDF]

Supplementary Material

Neuroinflammation is not a prerequisite for diabetes-induced tau phosphorylation.

Judith M. van der Harg, Leslie Eggels, Silvie R. Ruigrok, Jeroen J.M. Hoozemans, Susanne E. la Fleur and Wiep Scheper*

*** Correspondence:** w.scheper@vumc.nl

Supplementary Figure 1

Supplementary Figure 1

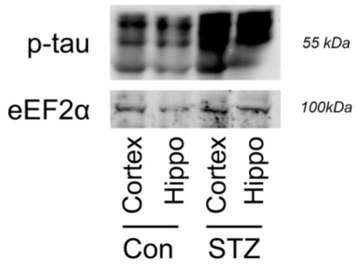

Increased tau phosphorylation at Ser396 in STZ-treated rats.
Representative western blot analyses of cortex and hippocampus (Hippo) protein lysates of rats 20 days after injection of citrate buffer (con) or STZ is shown. STZ-treated rats have increased levels of tau phosphorylated at Ser396 (p-tau). eEF2α is used as a loading control.
